# Toothache: Its Diagnosis and Treatment

**Published:** 1893-02

**Authors:** A. V. Elliott


ARTICLE VI.

### TOOTHACHE: ITS DIAGNOSIS AND TREATMENT.

BY A. V. ELLIOTT.

The first and most important thing to consider when a case is brought before our notice is the diagnosis. To the patient who comes for relief, whatever the cause, it is to him simply a toothache and nothing more, and that is sufficient. But to us as diagnosticians it is of importance to know for a certainty the cause; whether, for example, it arises from irritation of the pulp of the tooth from exposure, or of the periodental membrane, or because of some obscure pathological condition elsewhere. The diagnosis is not always easy. Ordinarily, when the patient presents for relief, we have little difficulty in discovering it. We find the cavity, and within it a pulp in a state of irritation; the proper remedy is applied, and the patient goes gratefully away. But sometimes we are embarrassed. There are crowns in every state of disintegration; root canals in all stages of putrescence and the development of gases.

Again, there may be no open cavities, but many teeth filled; no appearance of anything wrong, yet the patient suffers. The pain may come from exposure or near exposure of the pulp through a prolongation of a cavity not apparent to the eye, or the pain may arise from a metal filling placed too close to the pulp without adequate protection, or it may be developed from incipient inflammation about the root of a dead tooth. Impacted wisdom teeth are frequently painful to the patient, or it may be osseous deposits in the pulp chamber and canals; or the pain might have its origin somewhere else, and is felt in a tooth through the reflex action of the nerves. Rheumatic, gouty, hysterical, and neuralgic people, and women during gestation are subjects of these irregular phenomena. It may be from different metals in contact, or near by, producing an electro-chemical action. Though this, perhaps, is more theoretical than real. I have never seen such a case.

In reducing diagnosis and treatment to general principles, let us commence with the simplest. Fortunately for us, these cases of difficult diagnosis are rare. The patients who come to us for relief are usually suffering from either inflammation of the pulp or of the alveolo-dental membrane. My plan is to remove the *debris* and decalcified dentine in the cavity, being careful not to wound the pulp, and then fill with oxyphosphate, leaving enough of the decalcified dentine over the nerve to protect it, after first sterilizing it with bichloride of mercury. If the pulp is exposed, or nearly so, I make a medicated soothing pad of eugenol, oxide of zinc and a few threads of Japenese paper or cotton. This I place next to the pulp, and, after taking up the surplus moisture from the pad with absorbing paper, fill the cavity with soft cement with as little pressure as possible. A cement which does not knead up softly should not be used.

If the patient has a violent and jumping toothache, aching persistently, especially at night in bed, I devitalize the pulp. I first apply something to reduce the pain. I prefer eugenol, as it seems to leave the pulp in a receptive state for the arsenic. The latter I prefer to other agents, because it leaves the dead pulp in a better state for removal. At times we have neuralgia, but this usually disappears after direct treatment to the tooth.

Sometimes it is difficult to know if it is really a case of inflammation of the pulp, or if, it being dead, it is inflammation of the alveolo-dental membrane. The patient is feverish and apprehensive, and incapable of giving much assistance in the diagnosis. Test with cold water, watching the expression of the patient; if the pulp is alive there is generally no mistaking the fact, but it may be from an improper filling. Generally, where there is no marked indication there is no inflammation of the alveolo-dental membrane. We must search for cavities, and sound suspected teeth for tenderness. We must study the color of the gum, must interrogate the patient as to the symptoms, and when we find as a result of the examination that one tooth is more sensitive than others, and that the patient complains of a dull, heavy pain, and reports that the tooth seems to be longer than the others, and that drinking of hot liquids was painful, and cold not so, we may with confidence look for relief by boring into that tooth to give a vent to the confined gas. If the vent gives no relief by removing the pressure, we must "do all things to conciliate; failing in that, all things to crush." Toothache of this kind is one of the most distressing ills that human flesh is heir to, and owing to its situation and the nature of it, it is often difficult to give that speedy relief the patient longs for. Owing to the pain, and the absorption into the system of poison, constitutional disturbance is associated with the trouble.

The first thing indicated is to reduce the inflammation and prevent its further progress. If suppuration has not already advanced too far, blistering pads (I make mine of red pepper and cocaine, sewed up in little bags nearly covered on the surface with chlora percha, or strips of felt covered with wet gum tragacanth and pepper dusted over that; the gum softens in water, but does not dissolve), aconite and iodine, or strong iodine, can also be applied.

I recommend a hot foot bath, a dose of Epsom salts, and six-grain doses of antipyria, or apply leeches over the root. A lotion of cocaine, aconite, chloroform, and iodine, rubbed somewhat forcibly on the gum with the end of the finger, has a benumbing effect. Failing to get relief within a reasonable time by this, we must turn our attention to hastening the process of suppuration and the discharge of the pus. Hot poultices,* having two or three drops of landanum on each, should be applied, so as to assist the pointing to the right direction.

There are hopeless cases where the use of the forceps would be a mercy, and a possible insurance against much misery in the future. But judgment, qualified by honesty of purpose, should rule before the dentist yields to the suggestions of the patient and his own convenience, and extract a tooth that might be retained by proper treatment.

Erosion or wearing down of the crowns often gives rise to annoyance. The only thing to do is to cover with gold. Chloride of zinc gives temporary relief, and so does the nitrate of silver. But if the case be beyond filling, the nerve should be killed and removed. The same with erosion on the buccal sides of teeth, which is different from the other class. The solvent in this case must come from the mucous membrane directly opposite and in contact. Where all of the teeth seem to be sound, but their necks exposed and the gum in an unhealthy state, it is difficult sometimes to locate the pain. The patient himself is not sure of the tooth. Sounding gives no response, cold water sets all the teeth aching, as does cold air. Ordinarily, the pain passes away with the cause; at others it is persistent. The cause of it is exposure,—not enough covering over the cementum, which is not as thick over the nerve or as dense as dentine, and needs the further protection of bone and gum. Isolate the tooth giving the

---

* We presume the writer means on the gum. Poultices on the cheek are not admissible.—ED. "Items."

trouble. Bore into the tooth, inserting a little eugenol, sealing up with gutta percha. This method has the advantage also of testing the tooth as to its vitality. Sometimes, where the destruction of tissue is very great, the only thing to do is to kill the pulp, after being sure of the right tooth. Exercise of patience and perseverance. I have recently had experience in being misled by sympathy. I had a toothache which, I thought, was in the left upper cuspid. I rejoiced at the idea. I wanted to experience clinically, on myself, the different phases and symptoms of toothache. I did not ask for sympathy, which is not usually given to dentists, but when I was unable to find any cause for the pain, and it was growing unbearable, I consulted my friend, Dr. Schaffner, who, not finding any trouble with that tooth, commenced sounding all the teeth on that side, and when he reached the wisdom tooth I responded. Up to that time I had no suspicion that there was anything wrong with that tooth.

In doubt, do too little rather than too much; be governed by general principles, and treat topically.—*International.*

